# Postoperative Change in Ocular Torsion in Intermittent Exotropia: Relationship with Postoperative Surgical Outcomes

**DOI:** 10.1371/journal.pone.0162819

**Published:** 2016-09-13

**Authors:** Ju-Yeun Lee, Sungsoon Hwang, Shin Yeop Oh, Kyung-Ah Park, Sei Yeul Oh

**Affiliations:** Department of Ophthalmology, Samsung Medical Center, Sungkyunkwan University School of Medicine, Seoul, Korea; Rush University Medical Center, UNITED STATES

## Abstract

The aim of this study was to determine whether objective ocular torsion in intermittent exotropia (IXT) changes after recession surgery, and to evaluate the relationship between change in ocular torsion and clinical parameters in IXT. Sixty patients between 3 and 14 years of age underwent lateral rectus (LR) recession for IXT. Digital fundus photographs were obtained from both eyes of each subject and the disc-foveal angle (ocular torsion) was calculated using image software. We compared the preoperative and postoperative amount of ocular torsion, and analyzed the correlation between the difference in ocular torsion (DOC) and clinical parameters including age, duration of strabismus, stereoacuity, amount of preoperative exodeviation, and mean dose response. We categorized the patients according to DOC value: positive DOC value as group 1, and negative DOC value as group 2. A correlation between ocular torsion dominance and fixation preference was also investigated using the Kappa test. The mean ocular torsion was 15.8 ± 4.6 degrees preoperatively and 13.7 ± 5.1 degrees postoperatively. Compared with preoperative values, the mean ocular torsion showed a significant decrease after LR recession (p<0.001), and a greater preoperative ocular torsion was significantly associated with the amount of DOC (r = 0.37, p<0.001). Degree of stereopsis, mean dose-response, and postoperative exodeviation were significantly different between group 1 (positive DOC) and group 2 (negative DOC) (p<0.001, 0.030, and 0.001 respectively). The Kappa test showed that there was a significant correlation between the dominance of ocular torsion and fixation preference (p = 0.020). Therefore, change in ocular torsion after LR recession can be a useful supplementary indicator for evaluating the degree of fusional control and for predicting postoperative surgical response in IXT.

## Introduction

Ocular torsion is defined as an anatomic rotation around the anteroposterior axis of the eye. The assessment of ocular torsion is important to help understand the mechanisms of ocular motility abnormalities and to guide its management. Ocular torsion can be estimated by two methods: subjective and objective. These two types of ocular torsion are often inconsistent [1–3].

The change in ocular torsion is mainly determined by the oblique muscles. Thus, ocular torsion is mostly observed in patients with vertical strabismus including oblique muscle palsy and A type or V type strabismus [4]. However, it has been reported that ocular torsion can be observed in conditions other than cyclovertical strabismus. Some studies have demonstrated the major role of the rectus muscles in ocular torsion when associated with restrictions that occur in Graves’ orbitopathy or orbital wall fractures [5,6]. Other studies have revealed some degree of ocular torsion even in healthy individuals [7–10].

One recent study has reported that ocular torsion could be observed in intermittent exotropia (IXT) [11]. The amount of ocular torsion in IXT showed a significant relationship with severity of IXT, including the amount of exodeviation and the degree of stereopsis. Based on these observations, it can be expected that ocular torsion will change if the IXT conditions change. However, to the best of our knowledge, there has not been a study investigating objective changes in ocular torsion in IXT after surgery. Therefore, the aim of this study was to determine whether objective ocular torsion in IXT changed after recession surgery, and to investigate correlations with clinical parameters.

## Methods

This hospital-based retrospective observational study was a single-center study over a 2-year period from 2012 to 2014 that was conducted in accordance with the tenets of the Declaration of Helsinki. This study was approved by the ethics committee of the Samsung Medical Center Institutional Review Board. Patient records were anonymized and de-identified prior to analysis.

All subjects with a diagnosis of intermittent exotropia who underwent lateral rectus recession surgery were included. For study eligibility, the IXT had to meet the following criterion: exodeviation magnitude of 15 PD or more at distance and near, as measured by the prism and alternate cover test. Patients who had eyes with anatomical abnormalities, and those with a history of ocular surgery or other significant ocular diseases (oblique muscle palsy, congenital cataract, or other retinal disorders), alphabet pattern strabismus, vertical deviation, high myopia (refractive error > -6D), amblyopia, lateral incomitance of >5D, any neurologic deficit, or developmental delay were excluded from this study.

All patients underwent complete ophthalmic examinations including prism and alternate cover test, the fixation preference test, and Titmus stereo testing (Titmus Optical Co, Petersburg, Virginia, USA). Ocular alignment was tested by the prism and alternate cover tests, which were carried out at distances of 6 m in nine cardinal gazes and 30 cm in primary gaze. After ocular alignment testing, fixation preference testing was performed on all patients. Patients who wore spectacles kept them on during the testing. One experienced examiner (S.O) determined which eye was strabismic and which eye was fixating, noting if the patients spontaneously alternated between the two eyes or if one eye seemed to be preferred for fixation. If one eye seemed to be preferred for fixation, we characterized the patient as ‘right dominant’ or ‘left dominant’ depending on the direction of the fixating eye. If it seemed that the alternation between the two eyes was equal, we considered it ‘alternative fixation’.

We measured ocular torsion in both eyes using fundus photography. Fundus photographs were obtained by a fundus camera (Topcon Medical System, Tokyo, Japan) with the patients asked to look at an internal fixation target in order to align their eyes in the primary position. Both eyes were open and pupils were dilated during the examination. The images were taken by an experienced technician (Y.K). The disc-foveal angle was calculated from a single well-focused photograph using the National Institutes of Health image analysis software (ImageJ 1.42q; developed by Wayne Rasbands, National Institutes of Health, Bethesda, Maryland, USA). The detailed methodology for these measurements has been described in previous studies.^7,11^ We defined the disc-foveal angle as the angle between a straight horizontal line passing through the center of the optic disc and a line intersecting the fovea and the center of the optic disc. Each eye was measured separately. Two experienced examiners (J.L, S.H) who were blinded to the study results analyzed each image. To obtain a total amount of ocular torsion, we calculated ‘ocular torsion’ as a sum of each disc-foveal angle in both eyes of each subject. Extorsion was assigned as positive value, and intorsion as negative value for calculation. When extorsion was shown in both eyes, overall ‘ocular torsion’ was calculated as the sum of two disc-foveal angles in each eye. In contrast, when extorsion in one eye and intorsion in the other eye were shown in the fundus photograph, overall ‘ocular torsion’ was measured as the difference between two disc-foveal angles in each eye in order to avoid error due to head tilt.

Data on sex, age, duration of strabismus, best corrective visual acuity (BCVA), refractory error, fixation preference eye, preoperative angle of deviation (PD), preoperative stereoacuity, and the disc-foveal angle in both eyes were collected from electronic medical records.

We investigated the changes in ocular torsion after surgery, and also evaluated the relationship between the dominant eye of ocular torsion and fixation preference in IXT. We analyzed fundus photography in each subject and observed which eye had a larger degree of ocular torsion, which was defined as the ‘dominant eye of ocular torsion’. A difference between two disc-foveal angles < 0.1 was designated as the absence of dominance in ocular torsion.

In addition to this quantitative measurement of ocular torsion, we created two groups according to the difference in ocular torsion (DOC) between preoperative and postoperative measures. DOC was calculated as follow:

DOC=(preopT)−(postopT)

Where preopT is the amount of preoperative ocular torsion and postopT is the amount of postoperative ocular torsion (degrees).

We classified patients with positive DOC values into group 1, and those with negative DOC values into group 2 prior to analysis. Clinical parameters including the age, interval between onset and surgery, BCVA, preoperative angle of exodeviation, preoperative stereoacuity, and dose-response in recession surgery were compared between groups 1 and 2.

Statistical analyses were performed by an independent statistician. Data were analyzed using the Statistical Analysis System (SAS) (SAS Institute, Cary, North Carolina, USA). The intra- and inter-observer reproducibility values were analyzed using an intraclass correlation coefficient (ICC). Paired t tests were used to compare the mean preoperative and postoperative ocular torsion values, and Spearman’s correlation was used for analyzing the correlations between ocular torsion, DOC, and other clinical parameters including age, duration of strabismus, BCVA, preoperative angle of deviation, preoperative stereoacuity, and dose-response. Clinical parameters were compared between groups 1 and 2 with the Wilcoxon rank sum test and rank regression after adjusting for age, stereopsis, and preoperative angle of deviation. We also used the Kappa test to evaluate the relationship between the dominance of ocular torsion and the fixation preference of the eye. Statistical differences were considered significant when the P value was less than 0.05. Results are expressed as means ± standard deviations.

## Results

We enrolled 182 patients (364 eyes) included in eligible criteria. One hundred twenty two patients were excluded (70 had no ocular torsion preoperatively, 10 had vertical strabismus, 2 had a history of previous strabismus surgery, and 12 did not undergo fundus photography). Therefore, total 88 patients (176 eyes) including 41 males and 47 females were finally included in this study. The mean age was 6 ± 3 years (range, 3 to 14 years), and the mean postoperative follow-up was 1.9 ± 1.2 years. Seventeen cases of unilateral LR recession and 71 cases of bilateral LR recession were included. The duration of strabismus was 20.6 ± 18.8 months (range, 4 to 117 months). The mean amount of correction was 6.8 ± 1.1 mm (range, 5.5 to 9.0 mm). The mean preoperative deviation in patients with exotropia was 25.5 ± 4.2 PD (range, 15 to 40 PD) and 2.3 ± 2.3 PD 6 months postoperatively. The mean dose response was 3.5 ± 0.9 PD/mm 6 months postoperatively.

### Change in ocular torsion after surgery

**Fig 1:**
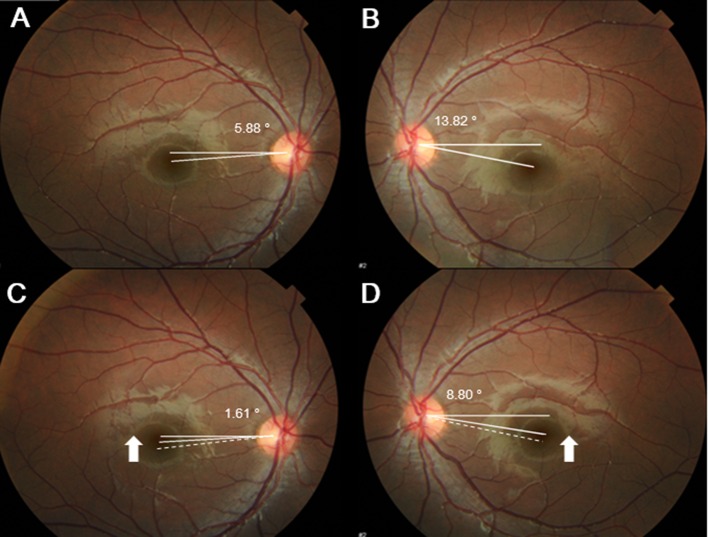
Change in ocular torsion after surgery. Compared with the preoperative condition, ocular torsion showed a significant decrease after lateral rectus recession in patients with intermittent exotropia,

Among the patients with ocular torsion in IXT, three patients (3.4%) showed exyclotorsion/incyclotorsion, 85 patients (96.6%) showed bilateral exyclotorsion, and no patients showed bilateral incyclotorsion in this study. There were no patients with subjective ocular torsion or head tilt in this study. The mean overall ocular torsion was 15.8 ± 4.6 degrees preoperatively, and 13.7 ± 5.1 degrees postoperatively. Compared with the preoperative values, the mean ocular torsion showed a significant decrease after LR recession (p<0.001) (Fig 1). The mean ocular torsion was 16.4 ± 4.2 degrees preoperatively, and 14.9 ± 4.3 degrees postoperatively in the unilateral LR recession group, and 15.7 ± 4.8 degrees preoperatively, and 13.4 ± 5.3 degrees postoperatively in the bilateral LR recession group. In each group, there was a significant decrease after surgery (p = 0.002, and <0.001 respectively). However, there was no significant difference in the amount of torsional change between the two surgical groups (Wilcoxon rank sum test, p = 0.792). Pilot study data of ocular torsion in patients who underwent LR recession surgery for IXT were presented in S1 Table.

### Correlation between ocular torsion, DOC and clinical parameters

**Fig 2:**
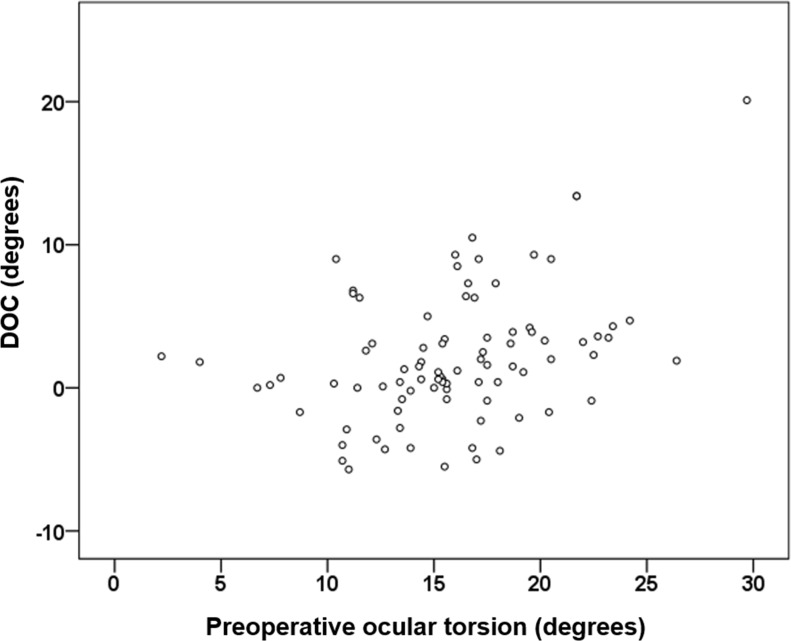
Correlation analysis showed that a greater preoperative ocular torsion was significantly associated with the amount of ocular torsion change (DOC) after lateral recession surgery (r = 0.37, p<0.001, Spearman’s correlation coefficient); DOC = Difference in ocular torsion between preoperative and postoperative value.

Correlation analysis showed that a greater ocular torsion correlated significantly with the amount of preoperative angle of deviation (r = -0.22, p = 0.038, Spearman’s correlation coefficient). A greater preoperative ocular torsion was significantly associated with the amount of DOC (r = 0.37, p<0.001, Spearman’s correlation coefficient) (Fig 2). However, other factors including age, duration of strabismus, stereoacuity, amount of preoperative exodeviation, and mean dose response were not significantly correlated with the amount of DOC (p = 0.883, 0.501, 0.794 and 0.644 respectively).

### The dominance of ocular torsion and the fixation preference

The results of this study also showed that 23 patients (26.1%) had right dominance in ocular torsion, 54 patients (61.4%) had left dominance in ocular torsion, and 11 patients (12.5%) showed an absence of dominance in ocular torsion. In the fixation preference testing, there were 32 patients (36.4%) with a right-fixating eye, 18 patients (20.5%) with a left-fixating eye, and 38 patients (43.1%) with alternative fixation. The Kappa test showed a correlation between dominance of ocular torsion and fixation preference (p = 0.020), with a Kappa value of 0.151 (95% CI, 0.017 to 0.285)

### A comparison of clinical parameters between the two groups

Among the participants, 62 patients (70.5%) were categorized into group 1 (positive DOC), and 26 patients (29.5%) were categorized into group 2 (negative DOC). Preoperative stereoacuity was 87 ± 85 arcsec in group 1 and 201 ± 160 arcsec in group 2. Surgical dose-response was 3.6 ± 0.9 PD/mm in group 1 and 3.3 ± 0.8 PD/mm in group 2. Postoperative exodeviation at 6 months was 1.6 ± 2.3 PD in group 1 and 3.9 ±1.3 PD in group 2. The degree of stereopsis, mean dose response, and postoperative exodeviation were significantly different between the two groups (p<0.001, 0.030, and 0.001 respectively, Wilcoxon rank sum test and rank regression) (Table 1). Other parameters including age, duration of strabismus, and amount of preoperative exodeviation were not significantly different between the two groups (p = 0.973, 0.559, and 0.469 respectively). The demographic data of the patients in this study are also listed in Table 1.

**Table 1:** Demographic data of patients diagnosed with intermittent exotropia and comparison of ocular torsion between the two groups (n = 88).

Variable	Group 1 (n = 62) Mean±SD	Group 2 (n = 26) Mean±SD	p Value
Age at surgery (months)	6±2	6±3	0.973*
Sex (male/female) (number)	30/32	11/15	0.643^a^
Duration of strabismus (months)	19.4±18.2	21.5±20.1	0.559*
Mean SE refractive error (diopters)	-0.73±1.84	-0.32±2.29	0.183*
Preoperative amount of ocular deviation (PD)	25.7±4.4	24.7±3.6	0.469*
Preoperative stereoacuity (arcsec)	87±85	201±160	<0.001*
Ocular torsion (degrees)	16.2±5.0	14.8±3.4	0.201*
DOC (degrees)	3.9±3.9	-2.8±1.8	<0.001*
Dose response (PD/mm)	3.6±0.9	3.3±0.8	0.030^
Postoperative amount of ocular deviation at 6 months (PD)	1.6±2.3	3.9±1.3	0.001^

* Wilcoxon rank sum test.

^a^ Fisher’s exact test.

^ Rank regression. SE, spherical equivalent; PD, prism diopters; DOC, difference value of ocular torsion.

Intra-observer reproducibility values were 0.96 preoperatively, and 0.96 postoperatively. Inter-observer variability values were 0.81 preoperatively, and 0.84 postoperatively. Excellent agreement was determined for intra- and inter-observer ICC reproducibility for all ocular torsion measurements. The study had an 80% or greater power to evaluate the difference in ocular torsion and DOC given the standard deviation of the ocular torsion and DOC measurements among the subjects in this study.

## Discussion

Ocular torsion is mostly observed in patients with cyclovertical strabismus. However, some authors have found that approximately one-third of patients with intermittent exotropia have ocular torsion. In a previous study by Shin et al. [11], the authors observed more frequent ocular torsion in patients with intermittent exotropia than in normal control subjects. They also found that the amount of torsion was positively correlated with the amount of preoperative exodeviation and the degree of stereopsis. No previous study has evaluated changes in ocular torsion after surgery and their clinical correlations. Thus, based on the results of these previous studies, we focused on the changes in ocular torsion after surgery in IXT.

This study demonstrated that ocular torsion in IXT was significantly decreased after LR recession. One previous study explained that ocular torsion in IXT might be derived from a change in the oblique muscles [12]. The authors reported that a persistent exotropic position could lead to the oblique muscles becoming slack and shortened, leading to a tonic imbalance of the oblique muscles. Ocular torsion in primary gaze was reported to be caused by this tonic imbalance of the oblique muscles. Thus, we can speculate that this tonic imbalance of the oblique muscles would be released after resolving exotropia by surgery, which could eventually lead to the release of ocular torsion in the primary position. Despite a statistically significant reduction, the quantitative amount of torsion decrease was so small that clinical significance is indefinite.

We also found that greater ocular torsion before surgery was significantly correlated with larger reductions in ocular torsion after surgery. This is supported by the work of Lee et al. [13], who observed that the amount of preoperative ocular torsion positively influenced the net change in ocular torsion after surgery in congenital superior oblique palsy. Although none of the participants in this study had any inferior oblique overaction, the tonic imbalance of the oblique muscles derived from a prolonged exotropic position could have a relatively consistent role in the change in ocular torsion in IXT.

The results of this study showed that ocular torsion dominance was significantly correlated with fixation preference in IXT. In the study by Olivier et al., the authors reported on the paradoxical cyclotorsion of the nonparetic eye in superior oblique muscle palsy [14]. They reasoned that this observation could be a result of a monocular sensory adaptation to cyclodeviation by a reordering of the spatial response of the retinal elements. Based on the results of this prior study, we expected that the dominance of ocular torsion would be consistent with the non-fixating eye. Despite statistical significance, however, caution should be used in interpreting this result since the kappa value was low (0.151). This finding contrasted with our expectations and is supported by the results of another study reporting the same objective torsion regardless of the eye used for fixation [12,15]. These authors found that there was no immediate torsional shift when the fixation switched. Our results indicate that the fixation preference in IXT does not significantly affect the motor correction for torsional misalignment.

In this study, no patients had subjective torsion. This finding is similar to Guyton’s observation that patients do not exhibit subjective torsion if their torsional problem developed in childhood [2]. All of the patients in our study were children, suggesting that IXT with tonic imbalance does not affect subjective perception of ocular torsion.

It is notable that patients who showed a reduction in ocular torsion after surgery had better stereoacuity. This result is in agreement with previous studies. Shin and associates reported that lower stereopsis was correlated with greater ocular torsion in IXT [11]. Graf et al. demonstrated that disrupting binocular fusion produced noticeable changes in excyclophoria [16]. They suggested that the torsional physiological position of rest is excyclo-rotated, and that the relative directions of this cyclophoria could appear to be independent after prolonged monocular occlusion. Because stereoacuity is one of the important indicators of the degree of fusional control in IXT, it seems reasonable to have presumed that patients with poor fusional control showed no reduction and might have even demonstrated an increase in objective ocular torsion in this study.

The degree of postoperative exodeviation and the dose-response effect of strabismus surgery for IXT require special attention with regard to their correlation with ocular torsion. Mean dose response was 3.6 ± 0.9 PD/mm in group 1, which was significantly higher than the mean dose response of 3.3 ± 0.8 PD/mm observed in group 2. The average difference between the two groups in surgical response was 0.3 ± 0.3 PD/mm. The mean degree of postoperative exodeviation was 1.6 ± 2.3 PD in group 1, which was significantly lower than the exodeviation of 3.9 ± 1.3 PD in group 2. Based on these results adjusted with other possible predictors, it is likely that patients with a reduction in ocular torsion after recession surgery in IXT have better surgical outcomes. The reason for these differences between the two groups is not clear. However, these findings can be adapted to predict surgical outcomes in IXT. We can assume that patients who show a reduction in ocular torsion after recession surgery would have better surgical outcomes compared with patients with an increase in ocular torsion after recession surgery.

There were several limitations to this study. First, we measured ocular torsion with fundus photography, which could have possible sources of error. Thus we cautioned against improper head position in order to minimize the effect of this error, and we also used the calculated value of total ocular torsion in each eye to adjust for the inevitable effect of head tilt in each subject. Second, we could not evaluate whether the fusion reduced or increased the ocular torsion. Further well-controlled studies are need to investigate it. Third, we only evaluated patients who underwent LR recession to compare the surgical effects under the same conditions. We found a significant relationship between the amount of ocular torsion and the long-term surgical effects in this study. Thus, further studies are needed that will investigate other types of strabismus surgery including recession/resection. Finally, we used a single center for this study, and all patients were of the same ethnicity. Thus, some of these results might not be valid for other ethnic groups.

In conclusion, this is the first study to demonstrate a significant change in ocular torsion after recession surgery in IXT, and described its clinical correlation. We found that larger reductions in ocular torsion after surgery were significantly associated with the degree of stereopsis and surgical response. The change in ocular torsion after LR recession surgery can be a useful supplementary indicator in the evaluation of the degree of fusional control and for prediction of long-term postoperative surgical response in IXT. Since we used surgical outcomes at postoperative month 6, further well-controlled studies are needed to assess the relationships between ocular torsion and long-term surgical outcomes.

## Supporting Information

S1 Table Pilot study data of ocular torsion in patients who underwent lateral rectus recession surgery for intermittent exotropia. (DOCX)
