# Supplementary material for: Postoperative Change in Ocular Torsion in Intermittent Exotropia: Relationship with Postoperative Surgical Outcomes
Source: PLoS One. 2016 Sep 13;11(9):e0162819. doi: 10.1371/journal.pone.0162819 (PMC5021304; doi:10.1371/journal.pone.0162819)
Supplement: S1 Table — (DOCX) [file pone.0162819.s001.docx]

| **Age** | **Sex** | **Fixation preference** | **Dominancy**  **of torsion** | **Preoperative**  **torsion** | **Postoperative**  **torsion** | **DOC** |
| --- | --- | --- | --- | --- | --- | --- |
| 5 | M | Alternative | Left | 22.5 | 20.2 | 2.3 |
| 12 | F | Alternative | Right | 16.1 | 7.6 | 8.5 |
| 8 | M | Alternative | no | 17.1 | 8.1 | 9.0 |
| 5 | F | Right | Left | 15.6 | 16.3 | -0.7 |
| 12 | F | Right | Left | 21.7 | 8.4 | 13.3 |
| 4 | F | Alternative | no | 22.4 | 23.3 | -0.9 |
| 8 | M | Alternative | Left | 20.2 | 16.9 | 3.3 |
| 9 | M | Left | Right | 19.6 | 15.7 | 3.9 |
| 11 | F | Alternative | Left | 14.7 | 9.7 | 5.0 |
| 7 | M | Left | Right | 15.4 | 12.3 | 3.1 |
| 9 | F | Right | Left | 10.7 | 15.8 | -5.1 |
| 6 | M | Alternative | Right | 15.6 | 15.8 | -0.2 |
| 14 | M | Alternative | Left | 15.5 | 12.1 | 3.4 |
| 6 | M | Right | Left | 24.2 | 19.6 | 4.6 |
| 8 | F | Left | Right | 17.9 | 10.6 | 7.3 |

**S1 Table.** Pilot study data of ocular torsion in patients who underwent lateral rectus recession surgery for intermittent exotropia

DOC=(preoperative torsion – postoperative torsion)
